# Infantile Thumb-Sucking

**Published:** 1875-10

**Authors:** 


Infantile Thumb-sucking.—*The British Medical Journal* publishes an article by Dr. Horacè Dobells, in which that distinguished physician states that he observed, that a peculiar and rather common deformity of the chest, is caused by the habit of sucking the thumb in infancy and early childhood. He says that the weight of the arm on the thorax of the child, during sleep, produces depression of the ribs in the line occupied by the arm, when the thumb is placed in the mouth. The doctor thinks this is a very important effect of the habit of thumb-sucking—one which has never before been pointed out—and he regards it of sufficient consequence to be put on record for the benefit of other observers.—*Pacific Med. and Surg. Journal.*

